# Feasibility study of ultrasound imaging for stereotactic body radiation therapy with active breathing coordinator in pancreatic cancer

**DOI:** 10.1002/acm2.12100

**Published:** 2017-06-02

**Authors:** Lin Su, Iulian Iordachita, Yin Zhang, Junghoon Lee, Sook Kien Ng, Juan Jackson, Ted Hooker, John Wong, Joseph M. Herman, H. Tutkun Sen, Peter Kazanzides, Muyinatu A. Lediju Bell, Chen Yang, Kai Ding

**Affiliations:** ^1^ School of Medicine Department of Radiation Oncology and Molecular Radiation Sciences Johns Hopkins University Baltimore MD USA; ^2^ Department of Mechanical Engineering Johns Hopkins University Baltimore MD USA; ^3^ Department of Computer Science Johns Hopkins University Baltimore MD USA; ^4^ Department of Ultrasound Zhejiang Cancer Hospital Hangzhou Zhejiang China

**Keywords:** intrafraction, pancreas, SBRT, ultrasound

## Abstract

**Purpose:**

Stereotactic body radiation therapy (SBRT) allows for high radiation doses to be delivered to the pancreatic tumors with limited toxicity. Nevertheless, the respiratory motion of the pancreas introduces major uncertainty during SBRT. Ultrasound imaging is a non‐ionizing, non‐invasive, and real‐time technique for intrafraction monitoring. A configuration is not available to place the ultrasound probe during pancreas SBRT for monitoring.

**Methods and Materials:**

An arm‐bridge system was designed and built. A CT scan of the bridge‐held ultrasound probe was acquired and fused to ten previously treated pancreatic SBRT patient CTs as virtual simulation CTs. Both step‐and‐shoot intensity‐modulated radiation therapy (IMRT) and volumetric‐modulated arc therapy (VMAT) planning were performed on virtual simulation CT. The accuracy of our tracking algorithm was evaluated by programmed motion phantom with simulated breath‐hold 3D movement. An IRB‐approved volunteer study was also performed to evaluate feasibility of system setup. Three healthy subjects underwent the same patient setup required for pancreas SBRT with active breath control (ABC). 4D ultrasound images were acquired for monitoring. Ten breath‐hold cycles were monitored for both phantom and volunteers. For the phantom study, the target motion tracked by ultrasound was compared with motion tracked by the infrared camera. For the volunteer study, the reproducibility of ABC breath‐hold was assessed.

**Results:**

The volunteer study results showed that the arm‐bridge system allows placement of an ultrasound probe. The ultrasound monitoring showed less than 2 mm reproducibility of ABC breath‐hold in healthy volunteers. The phantom monitoring accuracy is 0.14 ± 0.08 mm, 0.04 ± 0.1 mm, and 0.25 ± 0.09 mm in three directions. On dosimetry part, 100% of virtual simulation plans passed protocol criteria.

**Conclusions:**

Our ultrasound system can be potentially used for real‐time monitoring during pancreas SBRT without compromising planning quality. The phantom study showed high monitoring accuracy of the system, and the volunteer study showed feasibility of the clinical workflow.

## 1 INTRODUCTION

Pancreatic cancer remains one of the leading causes of cancer deaths in the United States.^1^ Currently, the only curable treatment option has been surgical resection. However, most patients are unresectable, as only 20% of patients are surgical candidates.^2^ For the unresectable patients, including the locally advanced and borderline resectable pancreatic cancer patients, the standard of care includes chemotherapy and radiation therapy. Although the optimal sequence, radiation technique, and total dose have not been well defined yet, recent advances in radiation therapy have improved the overall survival rate.^3^ Our institution experience has previously been reported to utilize definitive five‐fraction stereotactic body radiation therapy (SBRT) for locally advanced pancreatic cancer patients and borderline resectable pancreatic cancer patients. The report shows that chemotherapy of systemic gemcitabine followed by SBRT resulted in additional advancement toward optimizing patient outcomes.^4^, ^5^ Some patients even had margin‐negative resection and complete pathologic response with no remaining cancer cells found at the time of surgery.^6^

Despite our institutional pancreas SBRT experience, early radiation therapy studies likely had higher toxicity rates due to the lack of fractionation, inadequate motion management, lack of image guidance, and lack of specific dose constraints for organs at risk. The motion of the pancreas due to patient respiration is the primary source of intrafraction treatment uncertainties.^7^ Commonly employed motion management techniques include respiratory gating, active breathing coordinator (ABC), and abdominal compression.^8^ Due to the possibility that the stomach and duodenum may be pushed into the target volume, resulting in increased radiation toxicity to these structures, it is not recommended to use abdominal compression techniques. These methods physically restrict the abdominal muscle movement with either a plate or belt that applies a significant amount of pressure. In addition to motion management, intrafraction monitoring is becoming available in daily clinical use as it can verify the target location during the radiation therapy and thus eliminate the intrafraction treatment uncertainty due to motion, even under motion management techniques.^9^, ^10^, ^11^ Currently, several intrafraction motion monitoring methods have been developed. The predominant x‐ray‐based methods are limited either by the high level of imaging dose used for fluoroscopic imaging of small implanted markers or by the snapshot nature of the imaging data, such as those provided by cone‐beam CT (CBCT).^12^, ^13^, ^14^ The tracking of implanted electromagnetic transponders (i.e., Calypso) avoids the use of ionizing radiation but is unsuitable for pancreatic cancer given the large size of the transponder and the invasive procedure needed to implant them.^15^, ^16^

The recently introduced onboard MRI radiation systems offer a powerful real‐time, non‐invasive, and non‐ionizing solution to guide and monitor SBRT of soft‐tissue targets such as pancreatic cancer.^17^, ^18^ However, it remains uncertain as to whether these advanced and expensive systems will be generally available to the community. As an alternative, ultrasound imaging has low cost, the ability for image enhancement with contrast agents, mobility to be shared among machines, and compatibility to add to any existing treatment room.^19^, ^20^, ^21^, ^22^, ^23^ Ultrasound imaging has been previously developed for image‐guided radiation therapy and is commercially available for prostate intrafraction monitoring.^24^, ^25^ However, with the exception of recent efforts from the active robotic arm,^26^, ^27^, ^28^, ^29^, ^30^, ^31^ ultrasound imaging–based intrafraction monitoring clinical studies are still limited to prostate‐related applications. This is mainly due to the lack of probe holders for other sites such as pancreas and liver and the lack of treatment planning method to accommodate probe placement during treatment.^27^, ^28^

In this article, we introduce an arm‐bridge system for intrafraction real‐time motion monitoring during pancreas SBRT. We validated the image guidance workflow with volunteer study and studied the ultrasound monitoring accuracy using an ultrasound phantom and motion stage. We also investigated the impact of the probe placement in the treatment planning.

## 2 METHODS

**Figure 1:**
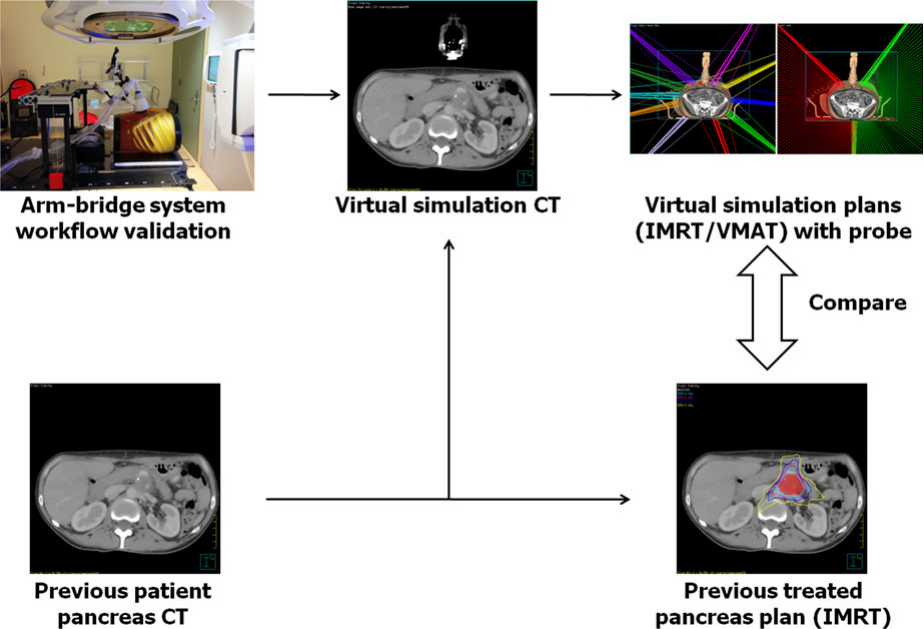
The study design workflow. The proposed arm‐bridge system CT was validated for image guidance workflow. It was segmented and fused with previous patient pancreas CT to create the virtual simulation CT. The IMRT and VMAT plans from the virtual simulation CT were then compared with the prior clinically treated pancreas IMRT plan.

Figure 1 shows a block diagram of the workflow of our study design. Our proposed arm‐bridge system was validated through the image guidance workflow. It was scanned and then segmented from the CT images. Previously treated pancreas SBRT patient CT images were fused with the segmented arm‐bridge system to create virtual simulation CT. Two types of treatment plans, both IMRT and VMAT, were generated by following our clinical pancreas SBRT protocol criteria and avoiding the probes in the virtual simulation CT. They were compared with the clinically treated pancreas SBRT with IMRT plans. In addition, a phantom study and volunteer study were performed to evaluate the accuracy of US monitoring. Participation of human subjects in the study was approved by the Internal Review Boards (IRBs) of the Johns Hopkins University School of Medicine where retrospective plans were analyzed and healthy volunteers were recruited.

### 2.A Arm‐bridge system

Our goal is to monitor the pancreas motion during the SBRT. Therefore, one of our top concerns for designing such system is its interference with treatment delivery. The optimal system should have the minimal blockage for planned radiation beam delivery. However, the desired probe orientation should allow the maximum scanning volume rate from the ultrasound probe. To accommodate these requirements, we designed the probe holder system as an arm‐bridge system. The system consists of a couch top bridge, articulated arms, infrared tracker, and ultrasound probe case. The bridge has rails on the bottom, enabling it to be attached to different couch tops. Two passive arms are used in the design to allow both fast placement and fine‐tuning of the probe position. Finally, a quick release mechanism on the probe case allows the user to detach the probe for freehand scanning.

**Figure 2:**
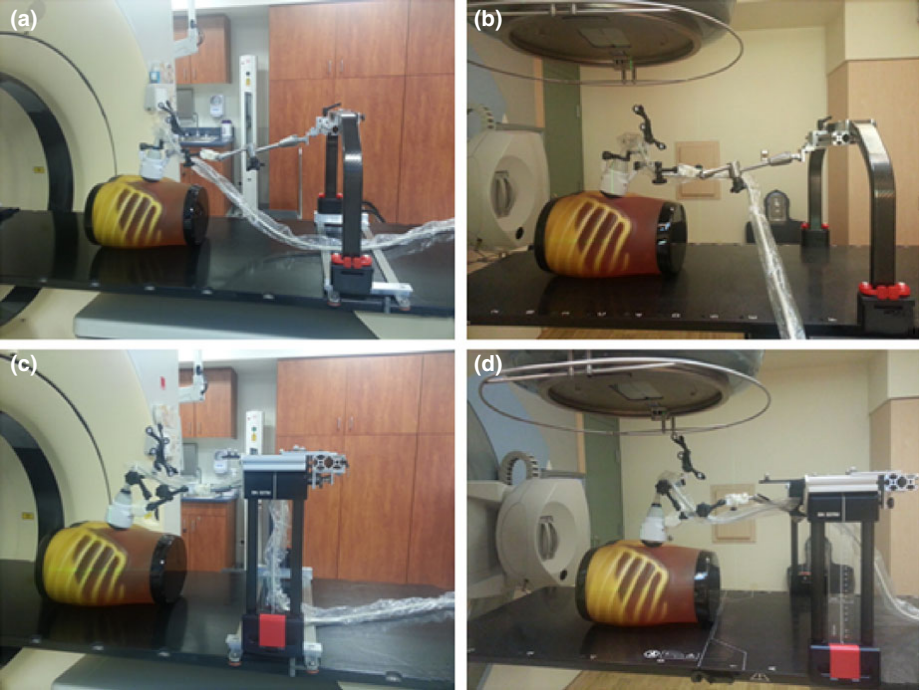
Ultrasound probe with the arm‐bridge system in simulation room and treatment room. (a) and (c) Arm‐bridge system for the Sim couch. (b) and (d) Arm‐bridge system for advanced HexaPod robotic couch top.

Figure 2 shows the proposed arm‐bridge system for both CT simulation room and treatment room. An ABDFAN ultrasound phantom (Kyoto Kagaku Co., Japan) is used in the setup. Figures 2(a) and 2(b) show our solution for simple simulation and treatment couch tops where an Elekta BodyFix frame can be installed as the bridge needed for the arm‐bridge system. Figures 2(c) and 2(d) show our solution for advanced HexaPod robotic couch top that utilizes an infrared camera tracked bridge (iGuide frame) for the couch position indicator. By adding an add‐on rail to the iGuide frame and using the long passive arm, our design eliminates the need for additional bridges and maintains the flexibility of the placement of HexaPod iGuide frame along the couch top. The bridge is secured on the couch top with an optional customized bottom rail. The bottom rails in Figs. 2(a) and 2(c) are designed for adapting the bridge to different types of couch tops such as the Varian couch top. Two double‐joint passive arms (long and short) attach the ultrasound probe to the aluminum rail mounted on the top of the bridge.

The long arm is used for quick adjustment of a general probe position, and the short arm is designed for fine‐tuning of the probe position and orientation. Both arms have knobs that secure the arm orientation and position once decided by the user. The end of the long passive arm can slide along an aluminum rail attached to the bridge, providing more flexibility for patient setup. A mechanically sweeping 4D convex probe (3–7 MHz) initially used in Clarity Autoscan (Elekta, Sweden) for prostate motion monitoring has been modified for our arm‐bridge system. The probe case has been designed to allow mounting of both a spider‐like infrared tracker (i.e., spider) and the short passive arm. The spider is tracked by the infrared camera mounted in the room. The probe is connected to the Clarity ultrasound acquisition system. The system is calibrated against the CT simulation room and treatment room isocenters to maintain the room coordinate consistency of the ultrasound image with the CT and CBCT images. After calibration, ultrasound image can then be fused to the planning CT in the Clarity image workstation. The short arm can be disconnected from the long arm from the adapter so that the user can operate the ultrasound probe freely during the initial scan. Once the optimal probe position and orientation is found by the user, the probe with the short arm can be connected back to the long arm. The user can then fine‐tune the position and orientations of the probe (i.e., roll, pitch, and yaw) using the short arm.

The time taken to acquire an ultrasound image volume depends on the imaging depth (probe axial direction), the number of lines or sector width (probe lateral direction), and the mechanical sweeping angle or number of frames (probe elevational direction). Based on our clinical experience, the major motion for the pancreas is in the patient superior–inferior direction. In our design, the ultrasound probe is oriented so that the mechanical sweeping or the elevational direction is aligned with the patient left–right direction to minimize ultrasound acquisition time and allow maximized volume scanning rate in future studies. The ultrasound probe lateral direction is aligned with the patient superior–inferior direction in the treatment room.

### 2.B Image guidance workflow validation

To validate our design in the setting of our pancreas patient image guidance workflow, we used the ABDFAN ultrasound phantom as mentioned in the previous section. After set up the phantom and the arm‐bridge system on CT couch top, we first localized pancreas in the ultrasound phantom and locked probe position with the arms under real‐time ultrasound guidance. Then, the ultrasound phantom was scanned with the Philips Brilliance Big Bore 16‐slice CT simulator using our clinical abdominal CT scan preset. Tumor LOC module was used to set the treatment isocenter in the scout CT image. Laser premarks and ball bearing markers were marked and attached to the surface of the phantom before acquiring the final scan of the planning CT. A 3D planning ultrasound image was obtained after aligning the phantom to the tumor LOC‐specified laser and couch position. The probe position and orientation were automatically tracked and recorded by the infrared camera and Clarity Sim software. The planning CT and ultrasound images were imported in Clarity AFC workstation for real‐time monitoring target planning. One of the simulated pancreatic tumors in the ABDFAN ultrasound phantom was contoured and set as real‐time monitoring target volume. The phantom and arm‐bridge system were then setup in the treatment room. To simulate patient motion, we placed the ultrasound phantom on a motion platform (Modus Medical, London, Canada). We aligned the phantom based on surface premark and acquired CBCT for couch shift by comparing CBCT to the planning CT. Ultrasound probe was then placed on the surface of the phantom. The placement is done by matching the recorded probe position and orientation using the interactive live guidance from Clarity Guide software. A 3D ultrasound image was then acquired, and the real‐time motion assessed by the monitoring module from Clarity Guide was recorded.

**Figure 3:**
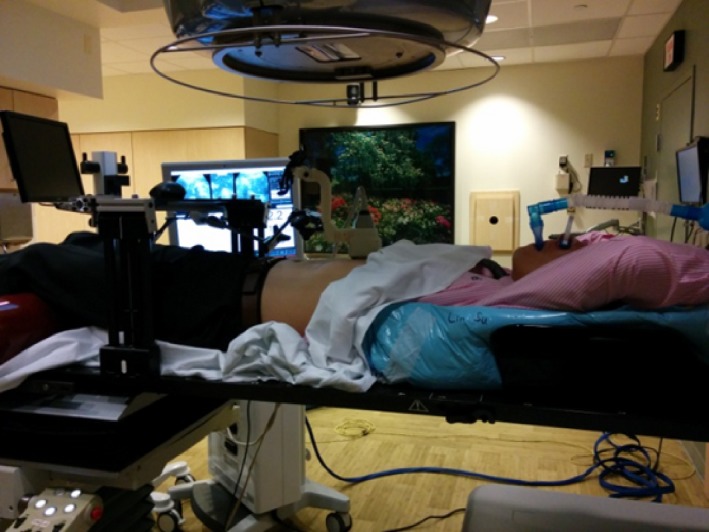
Volunteer setup in the treatment room with ultrasound system and ABC. The gantry rotated around the volunteer to assess clearance.

To further validate the clinical setting, we tested the ultrasound monitoring system with volunteer study, in which three volunteers of different sizes were included. The volunteer and ultrasound system were set up on CT couch, and the couch was moved through CT bore to test clearance. Then, volunteer and ultrasound system were set up on treatment couch with ABC. The gantry was rotated to different angles to check clearance (Fig. 3).

### 2.C Virtual simulation CT

To simulate the arm‐bridge system during CT scan and the planning, we created virtual simulation CT by combining the arm‐bridge system CT and previous patient simulation CT. We first setup the arm‐bridge system together with an ultrasound phantom and then scanned both of them with CT. The ultrasound probe, probe case, infrared tracker, and the short arm were segmented from the CT image and then virtually placed on the patient abdominal area in the virtual simulation CT by rigid fusion using VelocityAI software (Velocity Medical Solution, GA, USA). Both the patient's original CT and the virtual simulation CT have the same origin and coordinate, so the contours and treatment isocenter are identical. Ten patients with pancreatic tumors were randomly selected from the patients treated with SBRT at Johns Hopkins Hospital between 2014 and 2015. All ten patients had inoperable pancreatic cancer. The PTV volume ranges from 29.7 to 117.2 cm^3^, and the median PTV volume is 83.0 cm^3^. The patients include five males and five females. The patient age ranges from 41 to 79 yr, with a median age of 65.5 yr.

### 2.D Virtual simulation treatment planning

The clinically treated pancreatic SBRT plans in our institute used 10 or 11 coplanar IMRT beams, and ABC was used during simulation and treatment to constrain target movement. The plans were delivered by an Elekta Synergy linear accelerator with a HexaPOD robotic couch (Elekta AB, Stockholm, Sweden). The treatment planning was performed in Pinnacle v9.2 with direct machine parameter optimization. Several specific volumes were defined and contoured by our physician, namely the gross tumor volume (GTV), planning target volume (PTV), and organs at risk (OARs). These volumes were based on patient image datasets to facilitate treatment planning. GTV is contoured based on the CT and/or MRI images of the patient. A 2–3 mm expansion was made from GTV to PTV. The OARs include the radiation sensitive healthy organs and tissues close to the tumor. The OARs in pancreatic cancer typically include duodenum, stomach, bowel, liver, kidney, and spinal cord. The prescription of the pancreatic tumor is to deliver 33 Gy to PTV in five fractions.

For each virtual simulation CT, both IMRT and VMAT plans were created, following the same prescription and constraints as clinically treated plans. The beam angles were selected to be at least 30° away from the probe axis on both left and right sides. All control points were ensured to have the leaf end position in any opening MLC at least 3 cm away from the probe contour in the beam's eye view. For IMRT plans, 10 or 11 step‐and‐shoot beams were used, which were similar to clinical plans. The beams were distributed in the angles outside probe axis plus/minus 30°. The maximum number of segments was set to 70. The maximum of optimization iterations was set as 50. For VMAT plans, two dynamic arcs were employed. One arc covered from probe axis plus 30° to 180° in clockwise, the other arc covered from 182° to the angle as probe axis minus 30° in anticlockwise. The gantry spacing was set as 2° and maximum delivery time was set for 300 s. Plans with virtual simulation CT use the same objective or constraint as clinical plans for PTV and OARs from dose volume histogram (DVH). In addition, to achieve fast dose falloff of SBRT, after the first round of optimization, the regions of interest (ROIs) of 100% and 50% of prescription dose were generated. If the dose fall off did not pass the protocol, the ROIs of 100% and 50% of prescription dose were used as new objectives for a new round of optimization.

### 2.E Plan evaluation

All virtual simulation plans were required to pass our pancreas SBRT protocol dosimetric constraints as the clinical treatment plans. The parameters include GTV coverage, PTV coverage, and high‐dose volume of OARs. For target coverage, we evaluated dose to the PTV or Planning Target Volume. The target coverage was quantified as the percentage volume of PTV receiving dose larger than prescription dose 33 Gy (V33). Ideally, V33 for PTV should be 100%. For constraints on OARs, we evaluated the percentage of OAR volumes receiving a dose greater than 12 Gy (V12) in liver, kidney, and stomach, namely liver‐V12, kidney‐V12, and stomach‐V12. We also evaluated absolute OAR volume receiving dose larger than 15 Gy (V15) in the duodenum, stomach, and bowel, namely duodenum‐V15, stomach‐V15, and bowel‐V15. More details about these constraints defined by our institution can be found in a recent publication.^5^ In addition to the protocol, the following parameters were used to assess the plan quality: the minimal dose to 95% of the PTV (D95), minimal dose to 5% of the PTV (D5), mean dose to the PTV (Dmean), the conformal index (CI) which is the ratio between PTV and volume receiving dose larger than prescription dose, and homogeneity index (HI), which is the difference between D5 and D95, divided by Dmean. The mean and standard deviation of parameters listed here were calculated for clinically treated IMRT plan, virtual simulation IMRT plan, and VMAT plan. In addition, DVHs of clinically treated plan and virtual simulation plan were compared.

### 2.F Ultrasound monitoring accuracy and reproducibility

**Figure 4:**
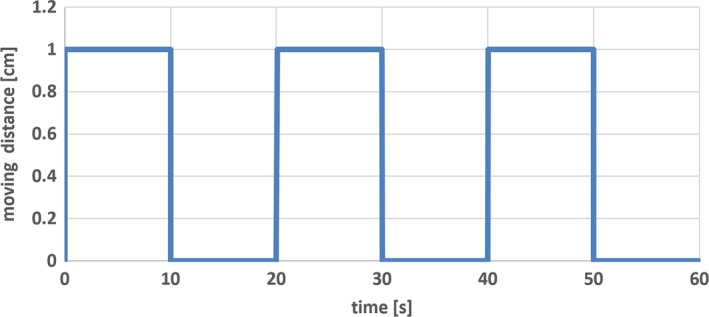
Graph showing simulated periodic breath‐hold movement of motion platform. The platform starts with one position (expiration), stays for 10 s, moves to another position (inspiration), stays for 10 s, and returns to the first position (expiration). The cycle could be repeated as many times as needed.

The ultrasound monitoring accuracy was tested with ABDFAN phantom and a motion platform. The phantom was secured on the platform. An optical tracker was attached to the phantom to provide the ground truth of phantom movement. The motion platform can be programmed to make a 2D movement. One side of the platform was elevated to create 3D movement. The motion platform makes simulated periodic breath‐hold movement. The stage starts with one position (expiration), stays for 10 s, moves to another position (inspiration), stays for 10 s, and returns to the first position (expiration). The movement curve of platform is shown in Fig. 4. One tumor inside the phantom was selected as tracking ROI. The ultrasound system was used to monitor the motion of tumor, and monitoring results were compared with the phantom motion tracked by the camera. As stated in Section 2.A, an optical marker is attached to the probe, which is captured by ceiling camera in real time. Any change of the probe position and orientation during phantom or volunteer movement can be detected immediately and will be accounted for in the monitoring.

In volunteer study, the reproducibility of ultrasound monitored ABC was investigated. The volunteers were guided to do ten breath‐holds with real‐time ultrasound monitoring. Superior mesenteric vein (SMV) was selected as monitoring ROI. The ultrasound imaging system performed real‐time monitoring based on ROI during ten cycles of breath‐holds. The positions of ROI for ten breath‐holds were recorded and compared to get the reproducibility.

## 3 RESULTS

### 3.A Image guidance workflow validation

**Figure 5:**
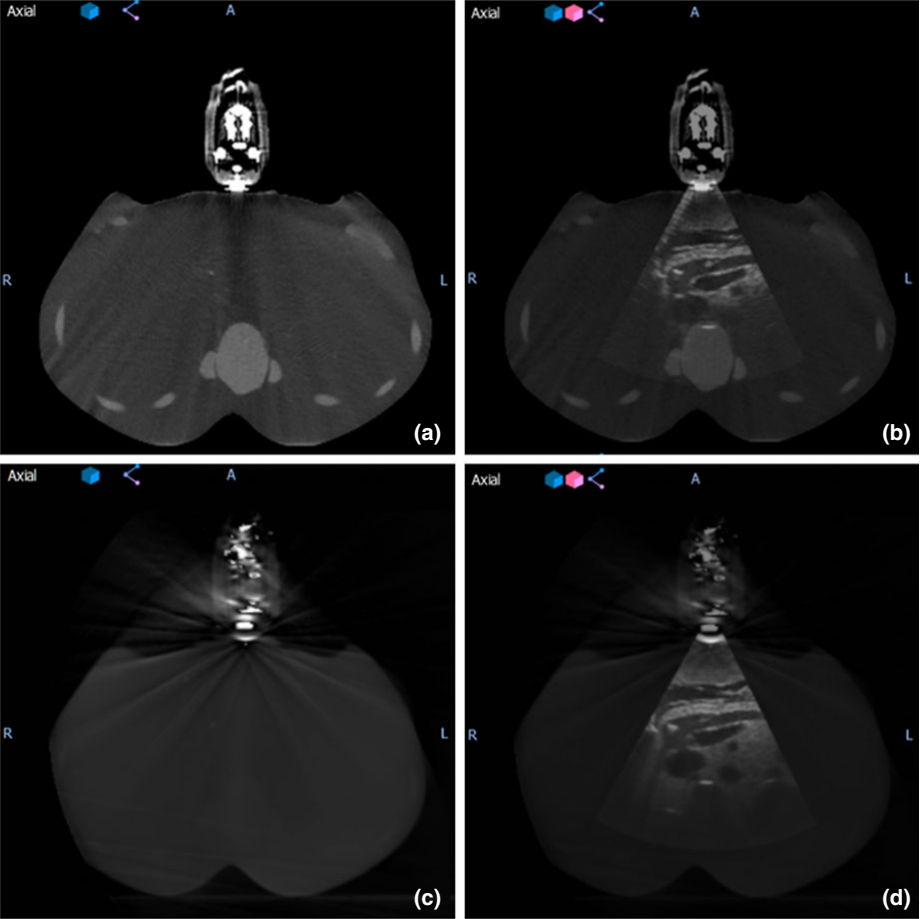
(a) CT image of the simulation setup, (b) fused ultrasound‐CT image of simulation setup, (c) CBCT image of the treatment setup, and (d) fused ultrasound‐CBCT image of treatment setup.

The arm‐bridge system and the ultrasound probe were validated in clinical image guidance workflow. Figure 5 illustrates examples of the transverse slices of CT, CBCT, and ultrasound images from the setup of arm‐bridge system together with ultrasound phantom as the setup described in Section 2.B. The CT and 3D ultrasound images in Figs. 5(a) and 5(b) were acquired in the CT simulation room for the pancreas area in the phantom. Figures 5(c) and 5(d) were obtained by CBCT and 3D ultrasound in the treatment room after aligning to the laser by premark on the phantom defined in the CT simulation room. Both ultrasound images show similar pancreas location in the room coordinate. The ultrasound phantom shows little contrast in CT or CBCT for the pancreas area but the high contrast in the ultrasound images. The ultrasound image acquisition system is calibrated to the isocenters of both simulation room and treatment rooms, which allows ultrasound‐guided interfraction setup as described in a recent review.^19^ The phantom left–right direction in the axial images of Figs. 5(b) and 5(d) corresponds to the ultrasound probe mechanical sweeping or the elevational direction. The patient superior–inferior direction corresponds to the ultrasound probe lateral direction.

**Figure 6:**
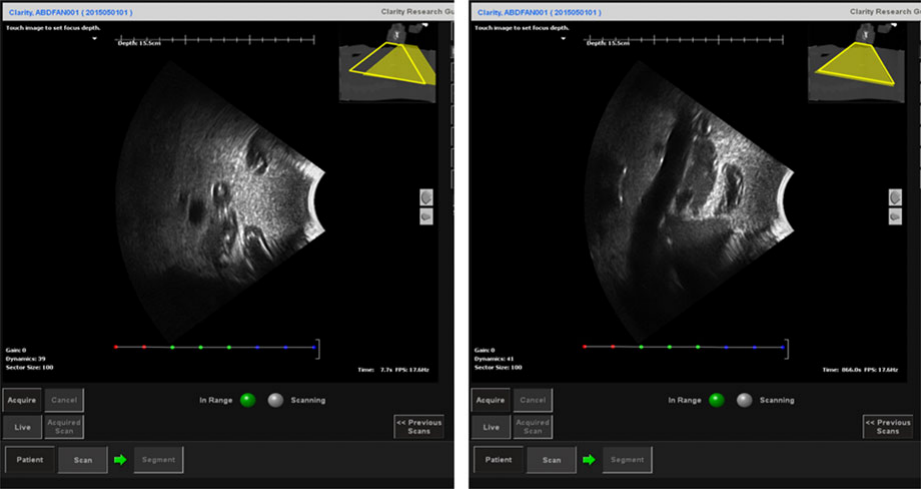
The sagittal view of the ultrasound image from the ultrasound phantom under the interactive guidance of probe placement before (left) and after (right) guidance. The trapezoids at the right top corner represent the real‐time probe position in Clarity Guide session (filled trapezoid) and recorded probe position in Clarity Sim session (empty trapezoid). The fused CT at the right top corner is the planning CT acquired during CT simulation.

Figure 6 shows an example of the ultrasound phantom under the interactive guidance of probe placement. The trapezoids at the right top corner represent the real‐time probe position in Clarity Guide session (filled trapezoid) and recorded probe position in Clarity Sim session (empty trapezoid). A mismatching between the two trapezoids represents a mismatching between the current probe position and the planned probe position. After matching the probe position, Fig. 6(b) shows a clear sagittal view of the pancreas from the ultrasound phantom which is similar to the ultrasound image acquired during simulation.

### 3.B Virtual simulation, treatment planning, and plan evaluation

**Figure 7:**
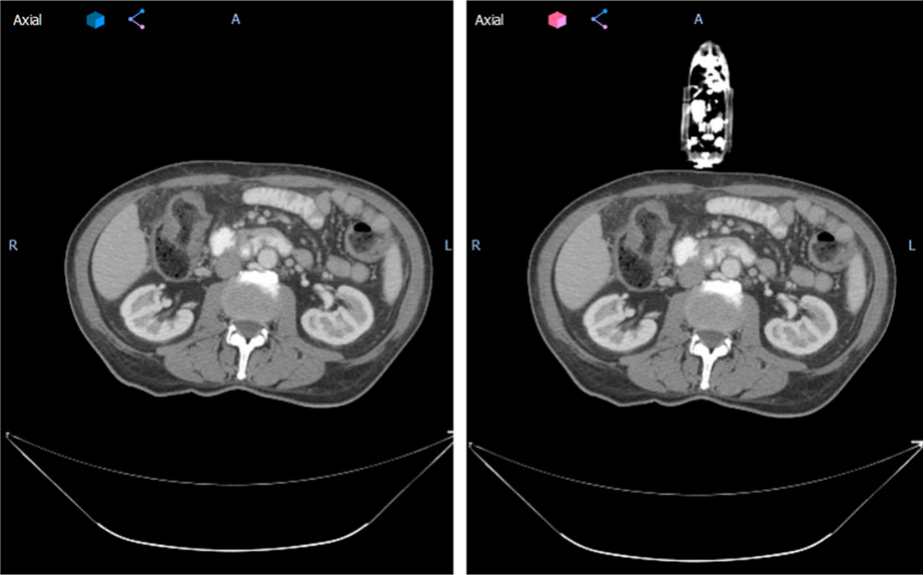
The axial view of an example patient from this study without (left) and with (right) virtual simulation probe. Patient left–right direction corresponds to the ultrasound probe mechanical sweeping or elevational direction.

After the setup of phantom and arm‐bridge system, a CT scan was performed for the whole setup as shown in Fig. 5(a). The results of virtual simulation are shown in Fig. 7, which includes an example of virtual simulation CT created for one of the previously treated patients. The arm‐bridge system components (i.e., ultrasound probe, probe case, infrared tracker, and short arm) were manually segmented from the CT image. A rigid fusion using VelocityAI was performed to fuse the above arm‐bridge system components to previous patient CT scan. The left part of Fig. 7 shows the original patient CT scan for the clinically treated plan. The result of the fused virtual simulation CT can be seen in the right part of Fig. 7.

**Figure 8:**
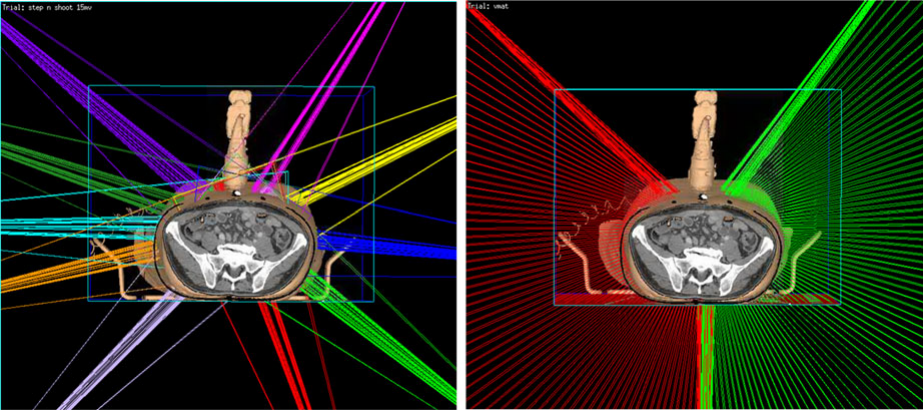
The 3D rendering of beam orientations for virtual simulation IMRT plan (left) and VMAT plan (right) of an example patient from this study in our planning system Pinnacle. The step‐and‐shoot IMRT plan consists of 10 beams, each beam has 5–10 segments. The VMAT plan is composed of two arcs; each arc has about 70 control points. Gantry angle interval between two consecutive control points is 2°. In both IMRT and VMAT plans, the beam is restricted as least 30° away from the probe.

Ten treatment plan were made on virtual simulation CTs. Figure 8 shows a case of beam arrangement of IMRT plan (left) and VMAT plan (right) from one of the patients with the virtual simulation CT. The 3D rendering of the ultrasound probe, probe case, and infrared tracker shows their relative location to the beam in the room. The patient was treated originally with ABC, an alpha cradle, and a wing board. The IMRT plan consists of ten beams, and the VMAT plan consists of two arcs.

**Figure 9:**
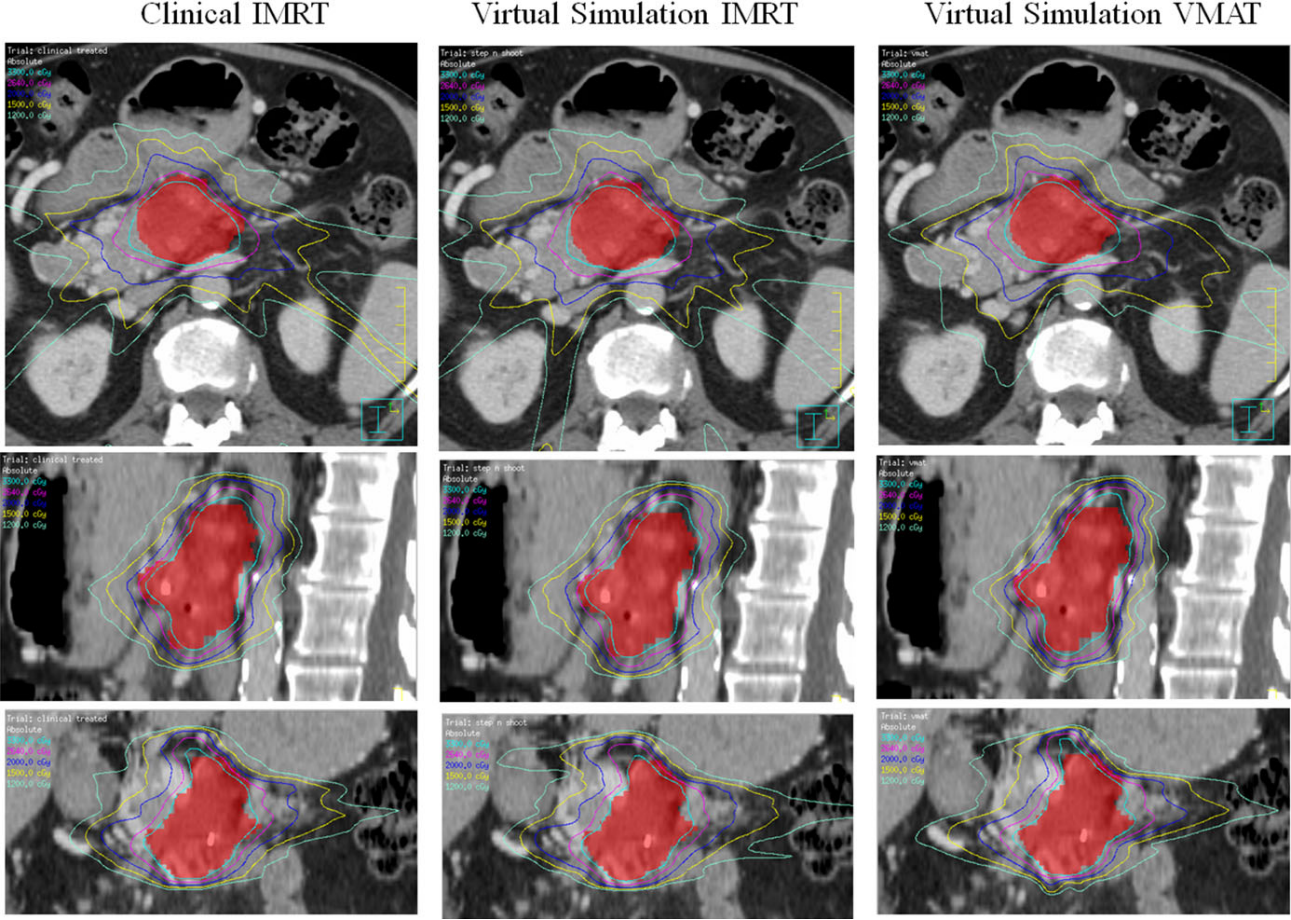
The isodose line comparison of different plans from one example patient. The left column shows the clinically treated IMRT plan. The middle column shows the virtual simulation IMRT plan. The right column shows the virtual simulation VMAT plan. The isodose line displayed are 3300 cGy (100% of prescription, in sky blue), 2640 cGy (80% of prescription, in purple), 2000 cGy (in blue), and 1500 cGy (in yellow). The V33 for PTV of clinically treated IMRT plan, virtual simulation IMRT plan, and virtual simulation VMAT plan are 92.9%, 93.3%, and 93.5%, respectively.

**Figure 10:**
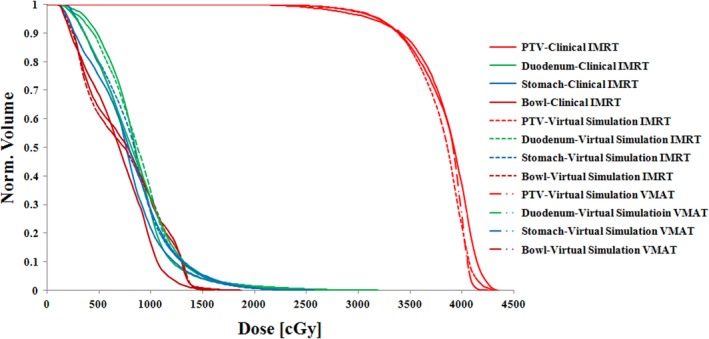
Dose–volume histogram (DVH) comparison of the clinically treated IMRT plan (solid lines), virtual simulation IMRT plan (dashed lines), and VMAT plan (dash‐double‐dot lines) from one patient in this study. The PTV coverage (V33) of clinically treated IMRT plan, virtual simulation IMRT plan, and virtual simulation VMAT plan are 92.9%, 93.3%, and 93.5%, respectively.

The virtual simulation plans generally achieved good dosimetric results. Figure 9 shows radiation isodose lines for different plan types. The left column is from clinically treated IMRT plan in our institution. The middle column is from virtual simulation IMRT plan. The right column is the virtual simulation VMAT plan result. The red color wash shows PTV in the pancreas area. The isodose line displayed are 3300 cGy (100% of prescription, in sky blue), 2640 cGy (80% of prescription, in purple), 2000 cGy (70% of prescription, in blue), and 1500 cGy (50% of prescription, in yellow). The V33 for PTV of clinically treated IMRT plan, virtual simulation IMRT plan, and virtual simulation VMAT plan are 92.9%, 93.3%, and 93.5%, respectively. This figure shows that the virtual simulation IMRT plan and VMAT plans with the arm‐bridge system and probe in placement can provide comparable dose conformity to PTV, compared with clinical treated IMRT plan. Figure 10 shows the dose volume histogram (DVH) of a patient in this study for three different plans. The solid lines represent clinically treated plan (clinical IMRT), the dashed lines represent IMRT plan from virtual simulation CT with the probe (virtual simulation IMRT), and the dash‐double‐dot lines represents VMAT plans with the probe (virtual simulation VMAT). The red lines are for PTV, green lines are for duodenum, blue lines are for stomach, dark red lines are for bowel. Three plans are comparable regarding DVH. The PTV of clinically treated IMRT plan, virtual simulation IMRT plan, and virtual simulation VMAT plan are 92.9%, 93.3%, and 93.5%, respectively.

All virtual simulation plans passed our institutional pancreas SBRT protocol criteria. Table 1 shows that for most of the PTV coverage parameters such as D95, V33, conformal index, and homogeneity index, differences between the clinically treated IMRT plan and the virtual simulation plans are not significant. For the Dmean and D5, the clinically treated IMRT plan shows larger deviation to the desired plan compared to the virtual simulation IMRT plans. However, this result is similar to the virtual simulation VMAT plan. For D95 and V33, the virtual simulation VMAT plan shows higher coverage than the virtual simulation IMRT plan. Based on OARs constraints (duodenum‐V15/20, stomach‐V15/20, liver‐V12, and stomach‐V12), as shown in Table 2, there is no major difference between the clinically treated plan and the virtual simulation plans. For kidney‐V12, clinically treated IMRT plan shows slightly more sparing to the kidney than the virtual simulation IMRT plan. For bowel‐V15, virtual simulation VMAT plan shows more sparing than the clinically treated IMRT plan. Detailed dosimetric parameters for all 10 patients are shown in Tables 3, 4, 5.

**Table 1:** Dosimetric parameters of PTV coverage from clinically treated IMRT plans, virtual simulation IMRT plans, and virtual simulation VMAT plans. D 95: the minimal dose to 95% of the PTV, D5: minimal dose to 5% of the PTV, Dmean: mean dose to the PTV, CI: conformal index, the ratio between PTV and volume receiving dose larger than prescription dose, HI: homogeneity index, the difference between D5 and D95, divided by Dmean

	Clinically treated IMRT	Virtual simulation IMRT	Virtual simulation VMAT
D95 [cGy]	3168 ± 121	3151 ± 78	3195 ± 80
Dmean [cGy]	3781 ± 63	3709 ± 63	3749 ± 82
D5 [cGy]	4122 ± 124	4020 ± 96	4043 ± 118
V33 [%]	92.8 ± 2.0	91.9 ± 1.4	93.3 ± 1.3
CI[ratio]	1.08 ± 0.07	1.03 ± 0.08	1.06 ± 0.09
HI [ratio]	0.25 ± 0.06	0.23 ± 0.03	0.23 ± 0.03

**Table 2:** Dosimetric parameters of OAR constraint from clinically treated IMRT plans, virtual simulation IMRT plans, and virtual simulation VMAT plans. V15 [cc]: volume of the structure receiving dose greater than 15 Gy in unit of cc, V20 [cc]: volume of the structure receiving dose greater than 20 Gy in unit of cc, V12 [%]: percentage volume of the structure receiving dose greater than 12 Gy

	Clinically treated IMRT	Virtual simulation IMRT	Virtual simulation VMAT
Duodenum‐V15 [cc]	5.66 ± 2.00	6.01 ± 2.17	5.24 ± 2.13
Duodenum‐V20 [cc]	1.19 ± 0.77	1.19 ± 0.69	1.17 ± 0.62
Stomach‐V15 [cc]	6.00 ± 2.54	6.12 ± 2.94	4.92 ± 2.45
Stomach‐V20 [cc]	1.02 ± 0.64	0.98 ± 0.65	0.96 ± 0.79
Bowel‐V15 [cc]	6.09 ± 2.05	5.78 ± 2.00	4.64 ± 1.99
Bowel‐V20 [cc]	1.27 ± 0.99	1.03 ± 0.98	1.44 ± 0.82
Liver‐V12 [%]	6.18 ± 5.01	7.02 ± 6.91	6.96 ± 7.06
Kidney‐V12 [%]	6.36 ± 6.65	7.08 ± 7.07	7.83 ± 7.55
Stomach‐V12 [%]	7.51 ± 4.26	7.01 ± 3.67	5.67 ± 3.30

**Table 3:** Dosimetric parameters of all ten virtual simulated IMRT plans. D 95: the minimal dose to 95% of the PTV, D5: minimal dose to 5% of the PTV, Dmean: mean dose to the PTV, CI: conformal index, the ratio between PTV and volume receiving dose larger than prescription dose, HI: homogeneity index, the difference between D5 and D95, divided by Dmean

Virtual IMRT plan	pt 1	pt 2	pt 3	pt 4	pt 5	pt 6	pt 7	pt 8	pt 9	pt 10	Mean	SD
D95 [cGy]	3227	3276	3082	3102	3026	3220	3235	3092	3111	3135	3151	78
Dmean [cGy]	3731	3667	3811	3693	3681	3802	3649	3649	3633	3770	3709	63
V33 [%]	93.4	94.3	91.2	90.7	91.1	93.3	92.3	90.1	90.5	92.2	91.9	1.4
Volume of 33 Gy + [cc]	35.6	129.2	87.2	84.0	112.2	50.1	53.4	108.9	42.3	101.6	80.4	31.3
Volume of PTV [cc]	29.7	117.2	86.9	81.2	104.1	50.1	57.4	115.0	44.3	95.8	78.2	29.4
CI [ratio]	1.20	1.10	1.00	1.03	1.08	1.00	0.93	0.95	0.95	1.06	1.03	0.08
D5 [cGy]	4001	4012	4157	4053	3958	4141	3892	3952	3893	4144	4020	96
HI [ratio]	0.21	0.20	0.28	0.26	0.25	0.24	0.18	0.24	0.22	0.27	0.23	0.03

**Table 4:** Dosimetric parameters of all ten virtual simulated VMAT plans D 95: the minimal dose to 95% of the PTV, D5: minimal dose to 5% of the PTV, Dmean: mean dose to the PTV, CI: conformal index, the ratio between PTV and volume receiving dose larger than prescription dose, HI: homogeneity index, the difference between D5 and D95, divided by Dmean

Virtual VMAT plan	pt 1	pt 2	pt 3	pt 4	pt 5	pt 6	pt 7	pt 8	pt 9	pt 10	Mean	SD
D95 [cGy]	3321	3300	3071	3200	3120	3228	3272	3121	3188	3132	3195	80
Dmean [cGy]	3815	3770	3825	3638	3683	3823	3743	3583	3818	3789	3749	82
V33 [%]	95.4	95.0	91.5	93.5	92.3	93.5	94.3	91.4	93.4	92.6	93.3	1.3
Volume of 33 Gy + [cc]	38.0	129.8	87.3	87.8	106.5	46.1	55.8	114.8	51.8	104.0	82.2	30.5
Volume of PTV [cc]	29.7	117.2	86.9	81.2	104.1	50.1	57.4	115.0	44.3	95.8	78.2	29.4
CI [ratio]	1.28	1.11	1.01	1.08	1.02	0.92	0.97	1.00	1.17	1.09	1.06	0.10
D5 [cGy]	4058	4148	4135	3898	3907	4097	4011	3842	4209	4129	4043	118
HI [ratio]	0.19	0.22	0.28	0.19	0.21	0.23	0.20	0.20	0.27	0.26	0.23	0.03

**Table 5:** Dosimetric parameters of all ten virtual simulated VMAT plans D 95: the minimal dose to 95% of the PTV, D5: minimal dose to 5% of the PTV, Dmean: mean dose to the PTV, CI: conformal index, the ratio between PTV and volume receiving dose larger than prescription dose, HI: homogeneity index, the difference between D5 and D95, divided by Dmean

Clinically treated plan	pt 1	pt 2	pt 3	pt 4	pt 5	pt 6	pt 7	pt 8	pt 9	pt 10	Mean	SD
D95 [cGy]	3295	3305	3092	3075	2960	3150	3307	3153	3301	3037	3168	121
Dmean [cGy]	3776	3729	3840	3780	3803	3855	3629	3756	3831	3813	3781	63
V33 [%]	94.9	95.1	91.1	90.0	90.0	93.0	95.2	92.6	95.0	91.0	92.8	2.0
Volume of 33 Gy + [cc]	36.7	123.5	94.9	79.4	111.5	57.9	60.4	120.8	48.5	100.1	83.4	29.7
Volume of PTV [cc]	29.7	117.2	86.9	81.2	104.1	50.1	57.4	115.0	44.3	95.8	78.2	29.4
CI [ratio]	1.23	1.05	1.09	0.98	1.07	1.16	1.05	1.05	1.09	1.05	1.08	0.07
D5 [cGy]	4028	4112	4167	4201	4215	4222	3797	4091	4182	4208	4122	124
HI [ratio]	0.19	0.22	0.28	0.30	0.33	0.28	0.14	0.25	0.23	0.31	0.25	0.06

In general, for most of the parameters including the PTV coverage and the three most important OARs (duodenum‐V15/20, stomach‐V15/20, and bowel‐V15/20), there are no significant differences between the virtual simulation plans and clinical plans. All virtual simulation plans pass the protocol requirement.

### 3.C Ultrasound monitoring accuracy and ABC

**Figure 11:**
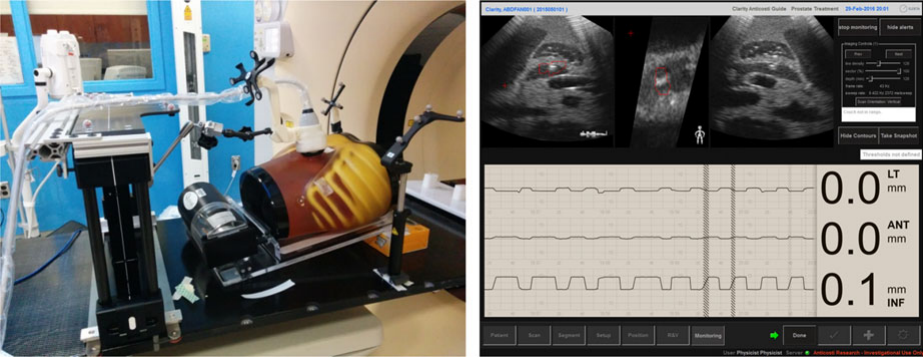
The ultrasound phantom, motion platform, and arm‐bridge system setup in the treatment room (left) and the Clarity real‐time monitoring of the ultrasound phantom motion (right). The monitoring module shows the 3D motion with time in left–right, anterior–posterior, and superior–inferior directions and real‐time ultrasound image views at the top row.

Our experiment proved good ultrasound monitoring accuracy of our system. Figure 11 shows the setup of ultrasound phantom, motion platform, and arm‐bridge system in the simulation room (left) and the Clarity Guide real‐time monitoring of the ultrasound phantom motion (right). After aligned to the laser with the premark on the phantom surface, a monitoring reference ultrasound image was acquired at the simulated exhale phase from the motion platform. The monitoring module then started to monitor the 3D motion with time in left–right, anterior–posterior, and superior–inferior directions and real‐time ultrasound image views. The phantom motion between inspiration and expiration captured by the camera (ground truth) is 2.35 ± 0.02 mm, 1.28 ± 0.04 mm, and 8.85 ± 0.03 mm in LR, AP, and SI direction, respectively. The motion monitored by ultrasound is 2.21 ± 0.07 mm, 1.32 ± 0.12 mm, and 9.10 ± 0.08 mm, respectively. The motion monitoring error in any direction is less than 0.5 mm. Detailed tracking results are shown in Table 6.

**Table 6:** Comparison between ultrasound‐monitored movement and movement tracked by camera. All numbers are in mm. LR: left and right direction, AP: anterior and posterior direction, SI: superior and inferior direction

Run ID	US tracking results			Camera tracked (ground truth)			Difference		
	LR	AP	SI	LR	AP	SI	LR	AP	SI
1	2.30	1.50	9.20	2.37	1.31	8.87	0.07	−0.19	−0.33
2	2.10	1.30	9.20	2.34	1.25	8.79	0.24	−0.05	−0.41
3	2.20	1.50	9.20	2.33	1.36	8.85	0.13	−0.14	−0.35
4	2.30	1.40	9.10	2.31	1.33	8.86	0.01	−0.07	−0.24
5	2.20	1.30	9.10	2.34	1.27	8.85	0.14	−0.03	−0.25
6	2.10	1.20	9.10	2.35	1.27	8.83	0.25	0.07	−0.27
7	2.20	1.20	9.00	2.35	1.24	8.84	0.15	0.04	−0.16
8	2.20	1.20	9.10	2.37	1.26	8.90	0.17	0.06	−0.20
9	2.20	1.20	9.00	2.35	1.28	8.87	0.15	0.08	−0.13
10	2.30	1.40	9.00	2.34	1.24	8.86	0.04	−0.16	−0.14
Mean	2.21	1.32	9.10	2.35	1.28	8.85	0.14	−0.04	−0.25
SD	0.07	0.12	0.08	0.02	0.04	0.03	0.08	0.10	0.09

In the volunteer study, the reproducibility of 10 ABC breath‐holds of all three volunteers was less than 2 mm. Detailed results can be found in Table 7. The data indicate our system could potentially provide accurate tracking of soft tissue in clinical settings.

**Table 7:** Movement monitored by ultrasound for 10 breath‐holds from three volunteers. All numbers are in mm. LR: left and right direction, AP: anterior and posterior direction, SI: superior and inferior direction

Run ID	Volunteer 1			Volunteer 2			Volunteer 3		
	LR	AP	SI	LR	AP	SI	LR	AP	SI
1	0.3	0.7	0.9	−1.7	0	−0.5	0	0.2	0.9
2	0.3	0.1	0.8	−1.9	−0.3	−0.4	0.1	0	1.1
3	1.2	1.3	1.4	−0.5	−0.2	0.4	−0.2	−0.3	0.6
4	0.2	0	1.6	−1	−0.2	0.4	−0.4	−0.2	−0.3
5	0.5	0.7	0.8	0	−0.3	−0.3	0.1	0.3	1.8
6	1.7	1.5	0.8	−0.3	0.6	0.7	1	1.2	1.5
7	0.2	0.2	0.2	−1.4	−0.2	0	0.6	1.1	1
8	0.1	0.1	0	−1	0.1	0.6	0.7	0.4	−0.3
9	1.4	0.6	1	−1.3	0.4	0.8	−0.2	−0.5	−0.6
10	0.1	0.4	1.5	0.1	0.2	0.9	1.5	0.4	0.3
Mean	0.60	0.56	0.90	−0.90	0.01	0.26	0.32	0.26	0.60
SD	0.60	0.51	0.52	0.70	0.31	0.52	0.61	0.56	0.81

## 4 DISCUSSION

While we are accumulating more clinical evidence to support the benefit of pancreatic cancer treated with SBRT, intrafraction treatment uncertainty due to motion may be potentially improved by using real‐time ultrasound monitoring. In addition to the similar advantage of being non‐invasive, non‐ionizing, and real‐time, ultrasound imaging as an alternative to MRI solution for abdominal soft tissue intrafraction monitoring, it can also potentially be added to most of the current existing treatment rooms rather than the need for a complete system replacement or new building as in MRI radiation systems. In this study, we designed and built an arm‐bridge system with bridge mounting rails and bottom adapter rails that can accommodate different couch types for real‐time ultrasound monitoring. The method has been evaluated in an abdominal ultrasound phantom in both simulation and treatment rooms with ultrasound imaging, CT, and CBCT. Moreover, the volunteer study demonstrated the feasibility of integrating this system into the current clinical workflow. The tracking accuracy in phantom study is 0.14 ± 0.08 mm, 0.04 ± 0.1 mm, and 0.25 ± 0.09 mm in the LR, AP, and SI directions, respectively. And the reproducibility of ABC for three volunteers are within 2 mm. By avoiding the delivery of IMRT and VMAT radiation beams through the ultrasound sound probe, we found that the planning quality is not compromised. It is, therefore, possible to achieve the same planning quality as the clinical plans when the probe and the arm‐bridge system are present.

However, there were several limitations to this study. Our design, mechanical clearance, imaging accessibility, probe stability, and deployment efficiency should be evaluated more comprehensively in a pilot study on patients. In addition, ultrasound imaging of the pancreas may not always be possible due to the bowel or stomach gas causing poor image quality. Patient education on diet at the initial consultation with nurses and physicians, and following diet restrictions are crucial to improving patient ultrasound imaging quality. During the phantom CT imaging in the simulation room and CBCT imaging in the treatment room, CT and CBCT images showed noticeable metal artifact from the probe as in Fig. 3. To mitigate the metal artifact from the probe, we have worked on strategies with promising results such as using a mock probe.^27^ Other groups also developed CT and CBCT reconstruction algorithms to reduce general metal artifact as in recent studies.^32^ A clinical implementation of metal artifact reduction algorithm from such studies can help us to improve the image quality result from CT and CBCT with an ultrasound probe in placement. The speed of sound correction is a known issue for the accuracy of ultrasound imaging. Several groups have studied the accuracy and potential impact on ultrasound imaging.^33^ In this study, we have not discussed the potential image degradation from the exit dose from radiation beam. It would be interesting to determine the dose level that can potentially degrade the image quality or damage the probe. In our study with the phantom and simulation CT, we did not include the soft‐tissue deformation introduced by the probe weight. Our future study with clinical patients will allow us to better understand the impact of the probe weight on soft‐tissue deformation, treatment planning, and ultrasound image quality.

## 5 CONCLUSION

Our ultrasound system can potentially be used for real‐time monitoring during pancreas SBRT. The phantom study showed high monitoring accuracy of the system, and the volunteer study showed feasibility of the clinical workflow from high reproducibility of the ABC breath‐hold. No planning quality compromise is required for pancreas SBRT treatment delivery with ultrasound imaging. Future studies will concentrate on the clinical trials with pancreas SBRT patients to optimize the clinical workflow for real‐time ultrasound monitoring with our arm‐bridge system.

## ACKNOWLEDGMENTS

The authors thank Dr. Martin Lachine and David T. Cooper for their inputs in the Clarity software. This work was supported, in part, by grants from the National Institutes of Health (NCI R01 CA161613) and Elekta to J. W.

## CONFLICT OF INTEREST

The authors declare no conflict of interest.
